# Seasonal changes in the body size of two rotifer species living in activated sludge follow the Temperature-Size Rule

**DOI:** 10.1002/ece3.1292

**Published:** 2014-12-02

**Authors:** Anna Kiełbasa, Aleksandra Walczyńska, Edyta Fiałkowska, Agnieszka Pajdak-Stós, Jan Kozłowski

**Affiliations:** Institute of Environmental Sciences, Jagiellonian UniversityGronostajowa 7, 30-387, Krakow, Poland

**Keywords:** Biological agent, bulking control, life histories, Monogononta, multivariate analysis, oxygen, process characteristics, Rotifera, temperature, wastewater purification

## Abstract

Temperature-Size Rule (TSR) is a phenotypic body size response of ectotherms to changing temperature. It is known from the laboratory studies, but seasonal patterns in the field were not studied so far. We examined the body size changes in time of rotifers inhabiting activated sludge. We hypothesize that temperature is the most influencing parameter in sludge environment, leading sludge rotifers to seasonally change their body size according to TSR, and that oxygen content also induces the size response. The presence of TSR in *Lecane inermis* rotifer was tested in a laboratory study with two temperature and two food-type treatments. The effect of interaction between temperature and food was significant; *L. inermis* followed TSR in one food type only. The seasonal variability in the body sizes of the rotifers *L. inermis* and *Cephalodella gracilis* was estimated by monthly sampling and analyzed by multiple regression, in relation to the sludge parameters selected as the most influential by multivariate analysis, and predicted to alter rotifer body size (temperature and oxygen). *L. inermis* varied significantly in size throughout the year, and this variability is explained by temperature as predicted by the TSR, but not by oxygen availability. *C. gracilis* also varied in size, though this variability was explained by both temperature and oxygen. We suggest that sludge age acts as a mortality factor in activated sludge. It may have a seasonal effect on the body size of *L. inermis* and modify a possible effect of oxygen. Activated sludge habitat is driven by both biological processes and human regulation, yet its resident organisms follow general evolutionary rule as they do in other biological systems. The interspecific response patterns differ, revealing the importance of taking species-specific properties into account. Our findings are applicable to sludge properties enhancement through optimizing the conditions for its biological component.

## Introduction

The Temperature-Size Rule (TSR) proposes a relationship between body size response and changing temperatures, and it has been documented for approximately 80% of ectotherms (Atkinson 1994). It states that organisms grow slower but for a longer time and achieve bigger size at lower temperatures, while the opposite is true at higher temperatures. This phenotypic, intraspecific response has a series of implications for performance and population growth because body size is the life-history trait most directly linked to the lifetime reproductive success (Kozłowski 1992; Roff 1992; Stearns 1992). The TSR puzzles researchers because, according to some theoretical models, organisms in favorable conditions (such as at relatively high temperatures) should grow larger and be rewarded by greater reproductive output, while those in harsh environments (such as at relatively low temperatures) should instead reach full size earlier instead of risking their life before reaching maturity (Kozłowski 1992; Roff 1992; Stearns 1992).

TSR was thought to be a nonadaptive limitation caused by different temperature thresholds for growth and development (van der Have & de Jong 1996; Walters & Hassall 2006; Forster & Hirst 2012), but its adaptive significance was also suggested in theoretical models (Angilletta & Dunham 2003; Kozłowski et al. 2004; Arendt 2011). Some adaptive hypotheses argue that temperature works only as an environmental cue, while the TSR responds to other related factors. One of the possible covarying factors is oxygen availability, which is known to decrease with temperature increase (Atkinson et al. 2006; Harrison et al. 2013). Thus, smaller organisms at higher temperatures would benefit by their more efficient distribution of oxygen to their cell or cells (Woods 1999; Frazier et al. 2001; Angilletta et al. 2004). The oxygen role in TSR has been proposed to be more important in aquatic environments (Verberk et al. [Bibr b700]; Forster, Hirst & Atkinson 2012) than in terrestrial ecosystems.

**Figure 1:**
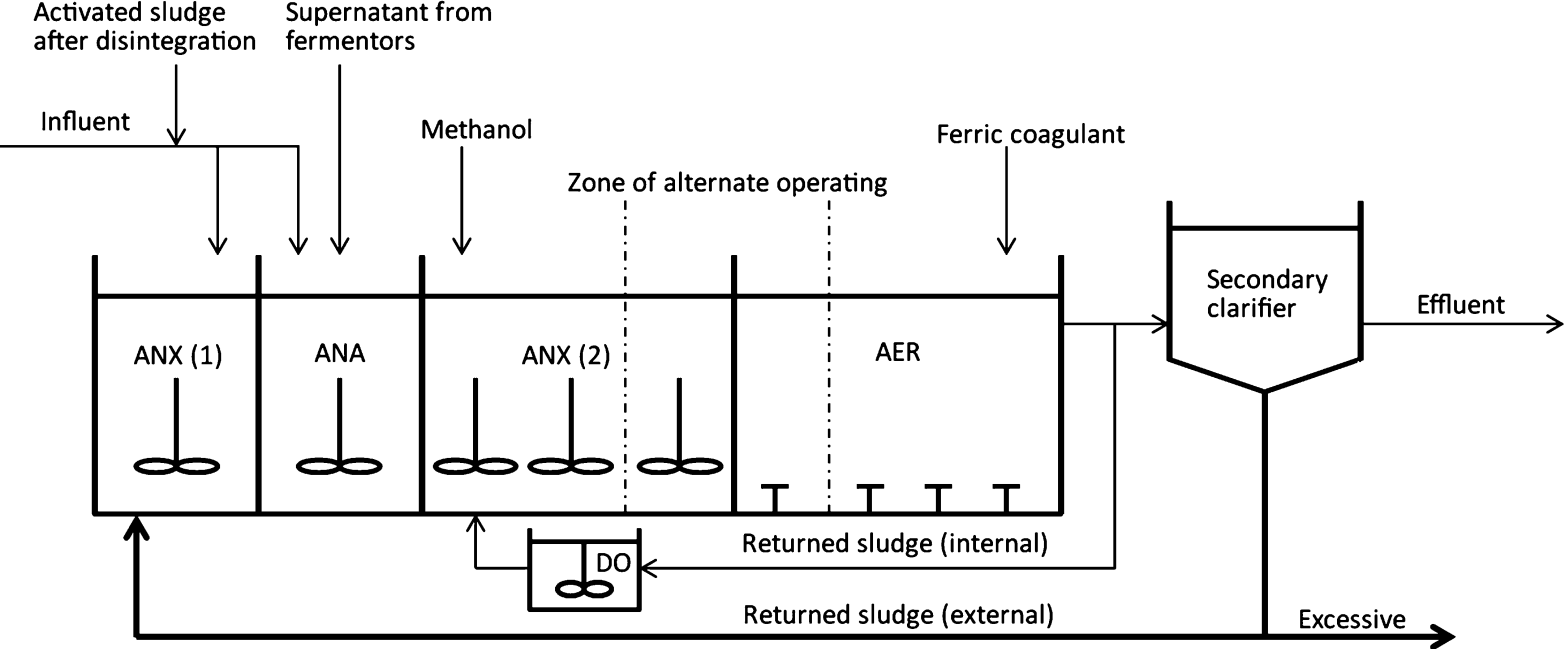
Diagram of the biological treatment zone in the Płaszów WWTP (modified from Pajdak and Łuszczek 2008). AER, aerobic nitrification chamber; ANA, anaerobic dephosphatation chamber; ANX (1), anoxic predenitriphication chamber; and ANX (2), anoxic denitrification chamber.

Wastewater treatment plants (WWTP) are promising systems for testing how body size responds to changing temperature and oxygen availability because the two variables are decoupled there; an automatic or manual regulation of the dissolved oxygen concentration in the aeration tank, maintaining levels sufficient for effective nitrification, is made regardless of the ambient temperature in activated sludge (Eikelboom 2000). Wastewater purification systems all over the world imitate the natural processes of organic matter decomposition, but under conditions that are artificially accelerated in the bioreactors of WWTP (Fig. 1). A dense mixture called “activated sludge” is used in the bioreactors, and it consists of flock-forming bacteria, protozoans that are flagellates, naked and testate amoebae, ciliates, and metazoans such as rotifers, nematodes, oligochaetes, and tardigrades (e.g., Bitton 2011). Activated sludge in wastewater treatment plants with nutrient removal is constantly inhabited only by certain specific types of organisms. They are resistant to higher loading of organic matter and ammonia than is found in the natural environment and are also able to proliferate in cyclically repeated anaerobic, anoxic, and aerobic conditions. Organisms in activated sludge are also constantly selected for higher population growth rates because organisms that proliferate slowly are removed from the system with excessive sludge. That is why only organisms with doubling times shorter than “sludge age” or “mean cell residence time” (MCRT) can survive in activated sludge systems.

The condition of an activated sludge is assessed by microscopic examination. When activated sludge is inhabited by rotifers, sessile, and crawling ciliates and testate amoebae, the flocks are firm and compact. The medium between flocks is clarified as appropriate. In a secondary clarifier (Fig. 1), the liquid–solid separation is undisturbed (Eikelboom 2000; Fialkowska et al. 2005). One of the frequent challenges of liquid–solid separation in WWTP is “bulking” as a consequence of the overproliferation of filamentous bacteria (Bitton 2011). Among numerous methods to control bulking in activated sludge, using organisms that are able to ingest filamentous bacteria is the most environmentally friendly. The most promising biological tool is the rotifer species *Lecane inermis* Bryce, which is naturally occurring in activated sludge (Klimowicz 1989; Fiałkowska and Pajdak-Stós 2008; Kocerba-Soroka et al. 2013). The rotifer *L. inermis* is not only able to improve sludge properties by reducing the number of filamentous bacteria but also to reduce biomass in bioreactors by grazing on the bacteria that are loosely attached to the flocks. In recent decades, research has focused on using the rotifers that are naturally occurring in activated sludge to limit the production of excess sludge (Lee and Welander 1996; Rensink and Rulkens 1997; Lapinski and Tunnacliffe 2003; Ratsak and Verkuijlen 2006).

Currently, the factor that limits the use of rotifers as a biological tool to control bulking is their temperature-dependent growth rate. As Fiałkowska et al. (2011) showed, the growth rate of *L. inermis* at 20°C varied between 0.41 and 0.47 day^−1^, with a doubling time between 1.5 and 1.7 day^−1^. At 8°C, one of the clones almost failed to proliferate, and another exhibited a doubling time of 7.9 day^−1^. The bulking phenomena in the temperate zone are mainly reported in winter and early spring, when temperature in bioreactors drops below 15°C (Eikelboom 2000; Jenkins et al. 2004). At these temperatures, there is a risk that *L. inermis* rotifers will be removed with excessive sludge. Pajdak-Stós and Fiałkowska (2012) hypothesized that bulking is more severe in certain seasons because the activity level of organisms that graze on filamentous bacteria depends on the temperature. Detecting the physical parameters that influence the body size of *L. inermis* and the proliferation of its population will help to parameterise the optimal settings for this important biological agent.

We pose two questions: (1) Does the rotifer *L. inermis* adjust its body size to seasonal changes in temperature or the oxygen content in activated sludge? and (2) Which sludge parameters are dominant in our study system? In addition, we experimentally determined the most suitable measure of *L. inermis* body size in a laboratory for question (1). We used two temperature regimes, so we were able to test for TSR under laboratory conditions. To realize task (2), we analyzed the relationships between sludge parameters using a multivariate analysis (PCA). This analysis was designed to confirm that temperature and oxygen were not correlated in our study system, as well as to examine whether there were any other sludge parameters that could interfere in the body size response to temperature and oxygen. The multivariate analyses were previously used to describe the sludge environment by Elissen et al. (2008). However, their study was large-scale and focused on worm population dynamics as related to spatial and temporal patterns in different wastewater treatment plants. Finally, we found that another rotifer species, *Cephalodella gracilis* Ehrenberg, was similarly abundant in activated sludge samples, and we tested whether the same environmental factors underlie the body size of both species.

In this study, we found that two rotifer species living in activated sludge adjusted their body size seasonally in response to temperature in accordance with the Temperature-Size Rule and that one of them also responded to oxygen changes, corroborating an important hypothesis that oxygen is the ultimate factor behind TSR. These results are applicable to the biological control of processes that take place in activated sludge. Additionally, in a laboratory study, *L. inermis* rotifer was found to perform TSR depending on food type.

## Materials and Methods

### Study site and species

The study was conducted in the “Płaszów” WWTP in Krakow, Poland. A diagram of its biological treatment zone is shown in Figure 1. The WWTP works as a plug-flow, low-loaded system with controlled relationship between biological oxygen demand (BOD) and mixed liquor suspended solids (MLSS) of 0.15–2.0 kg BOD × kg MLSS × day^−1^. Retention time is maintained between 7.8 and 16 h, and mean cell residence time (MCRT) (commonly known as sludge age) is between 10 and 16 days. After an initial mechanical treatment, waste water in this facility goes to the primary clarifier and then to one of five bioreactors of *c*. 24,000 m^3^ volume each, where the process of biological purification takes place (Pajdak and Łuszczek 2008). All of the biological samples were taken from a nitrification chamber, from locations at the same distance from the oxygen sensor.

Both species investigated are bacterivorous monogonont rotifers, abundantly occurring in wastewater treatment plants (Klimowicz 1989; Radwan et al. 2004). The *L. inermis* female life cycle lasts about nine–ten days, and the first offspring appears at the second or third day (Miller, 1931). *Cephalodella gracilis* is tolerant to wide range of pH (Radwan et al. 2004). It is similar in size to *L. inermis*, but the body shape of these two species has been different, implying ecological dissimilarities between them. The LK6 clone of *L. inermis* was isolated from wastewater treatment plant in southern Poland in 2011 and is cultured since then at the Institute of Environmental Sciences, Jagiellonian University, Krakow, Poland. This clone was used in the laboratory study.

### Environmental parameters in activated sludge

The physicochemical and biological data for activated sludge during the period of the study were provided by the administration of Płaszów WWTP. Temperature (°C) and dissolved oxygen concentration (DO, mg O_2_/L; oxygen, hereafter) were monitored continuously, and daily mean values for the study period were obtained. During 2012, oxygen was maintained at a level of 2 mg/L from January until 23 May. After that, oxygen was adjusted to the level of ammonia, and the values began to vary. The following sewage and sludge analyses were performed weekly: mixed liquor suspended solids (MLSS; dry mass per 1 m^3^ of activated sludge (kg/m^3^)), Sludge Volume Index (SVI, the volume of 1 g of dry mass of sludge after 30 min of sedimentation), ammonia concentration (mg N-NH_4_/L), and nitrate concentration (mg N-NO_3_/L). Every two to five days, the pH of the sewage was measured. We were also provided with MCRT as estimated by calculations that were based on several measured parameters. However, because the frequency of this parameter was less than weekly and because of the considerable error associated with the estimate, MCRT was not included in the main analysis. Nevertheless, we discuss the possible influence of this important parameter.

A multivariate PCA was used to select the most dominant parameters in the environment. For the analysis, temperature, oxygen, and pH were aggregated to weekly means by combining three values from throughout the day, when sludge parameters were estimated (all sludge parameters were measured on the same day each week). Each of the seven variables was checked for normal distribution in accordance with the PCA assumptions. Only the oxygen content and ammonia had to be transformed (by the third and fourth root, respectively). Ammonia was still not normally distributed, but the analysis was robust because both the raw and transformed data had similar qualitative results, with only slight quantitative differences.

### Body size – laboratory study

**Figure 2:**
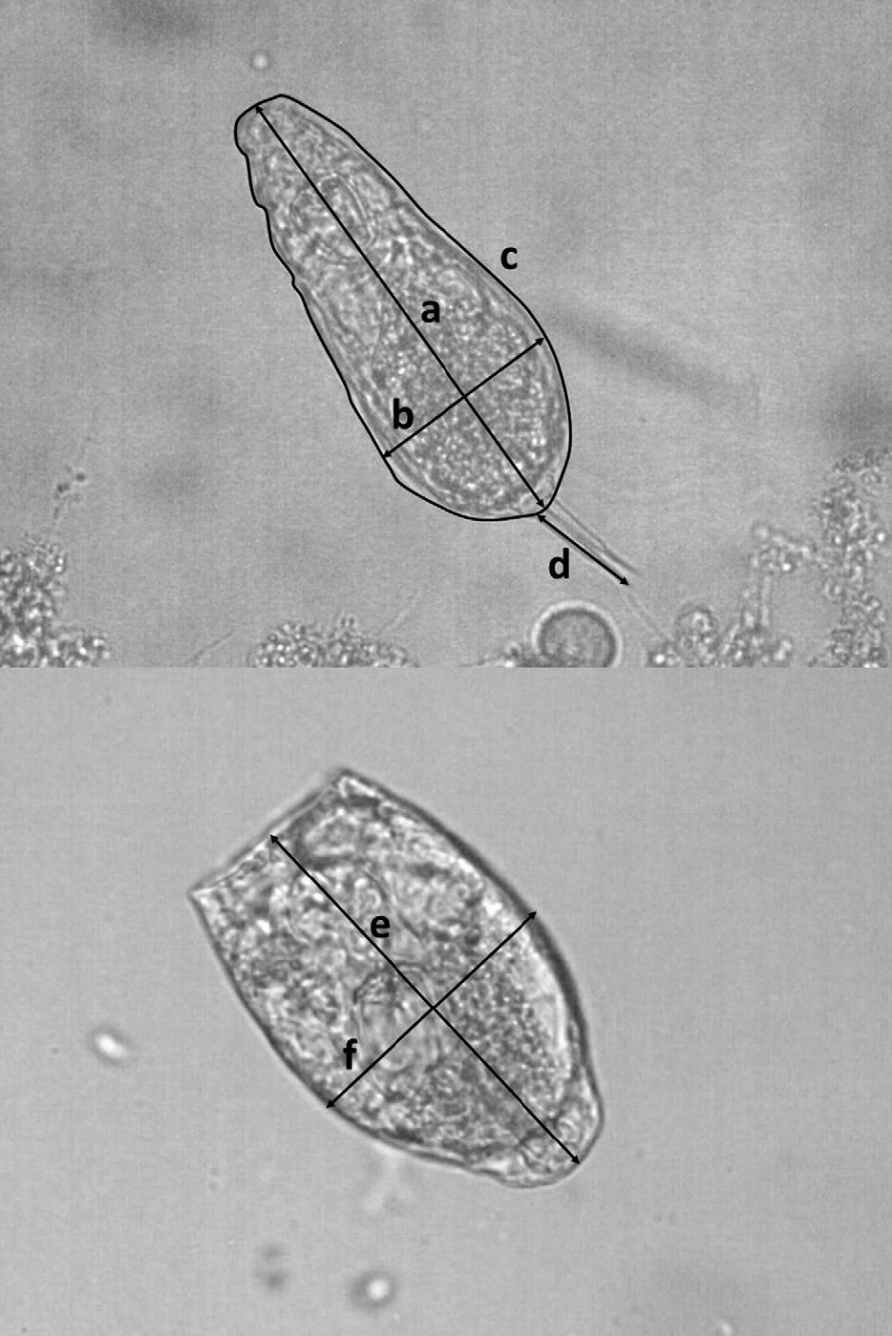
The five metrics measured to find the one most suitable for estimating body size response to temperature: (a) body length, (b) body width, (c) body area, (d) toe length, (e) lorica length, and (f) lorica width.

The performance curve for population growth rate of *L. inermis* at wide range of temperature (every 5°C between 10°C and 35°C) showed that 15°C was minimal temperature where population number increased (A. Walczyńska, unpublished data). Taking this result into account, a laboratory population of the *L. inermis* clone LK6 was divided into 15°C and 20°C treatments. Animals were cultured in Petri dishes with Żywiec Zdrój brand spring water as a medium. Food was provided ad libitum in the form of dried and sterilised yolk powder of two types: industrial dried (“ovopol”) or taken from hard-boiled eggs. After 3 weeks, *c*. 50 individuals per combination of temperature and food type were video-recorded (microscope Nikon Eclipse 80i, camera Nikon DS-U1 and PixeLink v. 3.2 Ottawa, Ontario, Canada software). Each short video sequence was then searched for the frame depicting the most outstretched individual. When possible, another frame with the same individual hiding within its lorica (protective covering) and with clearly visible toes was also selected. In order to find out the most consistent measure of rotifer size, the following metrics were measured (ImageJ, NIH, USA): body length, body width, body area (= contour), lorica length, lorica width, and toe length (Fig. 2). The dataset was restricted to individuals for which all five metrics were available (*N* = 48). The metrics were Z-standardized and checked for correlations. The results (Table 1) show that only toe lengths were uncorrelated with the other metrics and that body area was the most strongly correlated with the biggest number of other traits. Therefore, body area was selected as the best measure of size to detect the response to temperature, and only this metric was used in all further analyses. The differences in size in response to temperature (fixed) and food type (random) were taken into consideration and tested with a GLM model.

**Table 1:** Correlations of *Z*-standardized metrics of five measures of body size (*N* = 48). Bold values are significant at *P* < 0.05. Body area is the parameter most strongly correlated with all others, and therefore, it was chosen as a size measure in further analyses

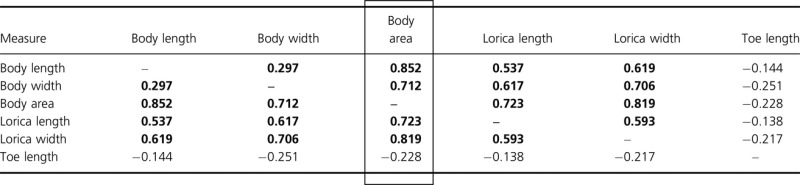

### Body size – sludge study

**Figure 3:**
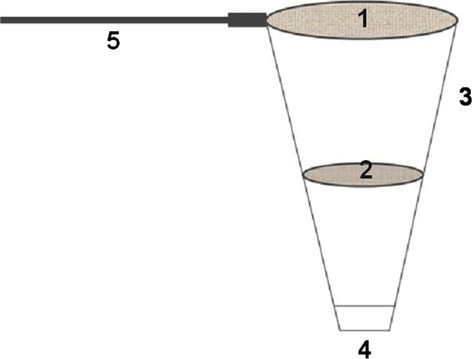
Diagram of the scoop for rotifer sampling. 1 – metal sieve with c. 250 *μ*m mesh (19.5 cm diameter); 2 – mesh sieve with holes of 32 *μ*m (12 cm diameter); 3 – planktonic mesh; 4 – outfall; and 5 – telescopic handle.

The sampling of rotifers was conducted from January until December 2012. Samples were taken once a month from the same place within an aeration tank (AER zone in Fig. 1), in the proximity of the oxygen sensor, by a two-sieve scoop (Fig. 3). Larger particles were retained on a sieve of mesh size ∼250 *μ*m, while rotifers were separated on a sieve of 32 *μ*m mesh. The smallest particles and microorganisms flowed through both sieves. After the scoop was dipped five times, the sample retained on the smaller sieve was rinsed into a Petri dish of 120 mm diameter. Three to five Petri dishes were sealed with parafilm and transported to a laboratory, then filtrated through a membrane of 120 *μ*m. The filtrate was searched for rotifer individuals. All individuals found were measured in the same way as in a laboratory study. To avoid measuring the new-born rotifers that had hatched under laboratory conditions, the entire procedure was performed on the day of sampling. Single specimens were placed on the glass slides. The most suitable size metric, selected in the laboratory study, the body area, was then recorded for measurements. A simple one-way ANOVA was used to analyze the seasonal variability of body size in each rotifer species, with month as an independent factor.

Another monogonont rotifer, *C. gracilis*, appeared similarly abundant in the samples, and we included it in our study beginning with the July sampling. We found no specimens of this species in December. In total, 88 individuals of *L. inermis* and 84 individuals of *C. gracilis* were measured. Multiple regressions were conducted to test the effect of temperature and oxygen content on the body sizes of each species separately. Two different analyses were conducted, with monthly means (= from 1 May to one day before the rotifer sampling in May and then from the day of the last sampling to the day before current sampling for all other months) and mid-monthly means (= a mean for the three values around a mid-period between rotifer samplings) of each independent variable. All analyses were performed in Statistica 10 (StatSoft). Figure 9 was created using CANOCO v. 5 (Ter Braak and Smilauer 2012).

## Results

### Environmental parameters of activated sludge

**Figure 4:**
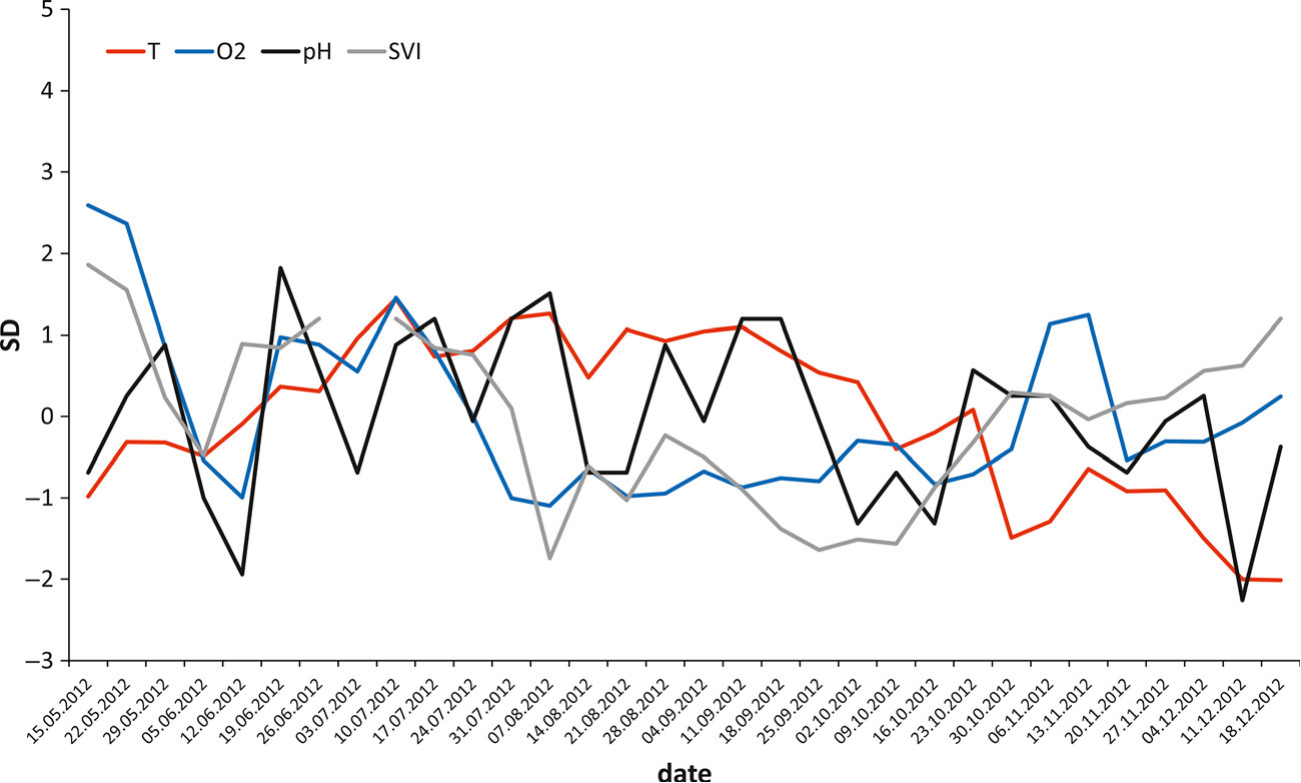
The *Z*-standardized values (weekly means) of the sludge parameters that most influence the sludge environment according to PCA analysis throughout the sampling period.

**Figure 5:**
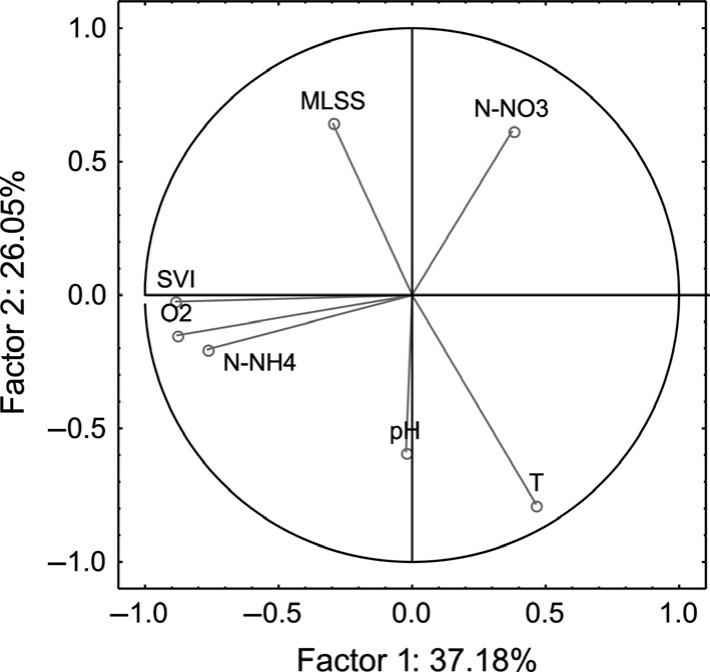
Associations among the sludge parameters (weekly means) on the factor-plane in PCA analysis.

The values of the sludge parameters we analyzed are summarized in Table 2. The correlation table (Table 3) shows the relationships among the examined parameters for *N* = 32 time points (weekly means). According to our assumptions, the two factors of greatest interest, temperature and oxygen content, are not correlated. Temperature is most positively correlated with pH and negatively correlated with MLSS, while oxygen content is positively correlated with SVI (Table 3). According to the PCA, the three factors with eigenvalues >1 explain 78.97% of the total variation. The factor loadings (Table 4) suggest that Factor 1 is driven by SVI and oxygen, Factor 2 is associated with temperature, and Factor 3 is associated with pH. Figure 4 shows their relative changes in units of SD (Z-standardisation) for the period May–December 2012. The associations among all of the parameters included in PCA analysis are illustrated in Figure 5. Because pH is significantly correlated with temperature and oxygen content with SVI (Table 3, Fig. 5), we analyzed the body size response of rotifers to temperature and oxygen only.

**Table 2:** The measured values of sludge parameters, provided by the Płaszów WWTP for the period of rotifer sampling (May–December 2012)

Parameter	Mean ± SD	Min. (month)	Max. (month)
T (°C)	18.6 ± 2.3	12.2 (December)	22.5 (July)
O_2_ (mg/L)	1.14 ± 0.43	0.66 (August)	2.29 (May)
pH	7.5 ± 0.2	7.0 (June, December)	8.0 (September)
MLSS (kg/m^3^)	3.97 ± 0.64	2.79 (August)	5.47 (July)
SVI (mL/g)	170.2 ± 45.5	89.6 (August)	253.0 (May)
N-NH_4_ (mg/L)	0.55 ± 1.02	0.01 (June)	4.98 (July)
N-NO_3_ (mg/L)	8.09 ± 2.50	1.44 (May)	12.0 (December)

**Table 3:** Correlations among the sludge parameters analyzed (*N* = 32). Values in bold are significant at *P* < 0.05

Variable	*T*	O_2_	pH	MLSS	SVI	N-NH_4_	N-NO_3_
*T*	–	−0.265	**0.474**	−**0.452**	−**0.375**	−0.009	−0.264
O_2_	−0.265	–	0.117	0.054	**0.701**	**0.437**	−**0.374**
pH	**0.474**	0.117	–	−0.068	0.015	0.236	−0.017
MLSS	−**0.452**	0.054	−0.068	–	0.235	0.040	0.289
SVI	−**0.374**	**0.701**	0.015	0.235	–	**0.402**	−**0.391**
N-NH_4_	−0.009	**0.437**	0.236	0.040	**0.402**	–	−**0.373**
N-NO_3_	−0.264	−**0.374**	−0.016	0.288	−**0.391**	−**0.373**	–

T, temperature; MLSS, mixed liquor suspended solids; SVI, Sludge Volume Index.

**Table 4:** Factor coordinates of the variables analyzed by PCA for the three factors with eigenvalues >1

Variable	Factor 1 (37.2%)	Factor 2 (26.0%)	Factor 3 (15.7%)
*T*	0.465	−**0.785**	0.137
pH	−0.023	−0.590	**0.750**
MLSS	−0.297	0.644	0.428
SVI	−**0.888**	−0.024	−0.087
N-NO_3_	0.376	0.616	0.523
O_2_	−**0.882**	−0.151	0.012
N-NH_4_	−0.769	−0.205	0.237

T, temperature; MLSS, mixed liquor suspended solids; SVI, Sludge Volume Index.

The percentage of variation explained is given in parentheses. Parameters most affecting each factor are bolded.

### Body size – laboratory study

**Figure 6:**
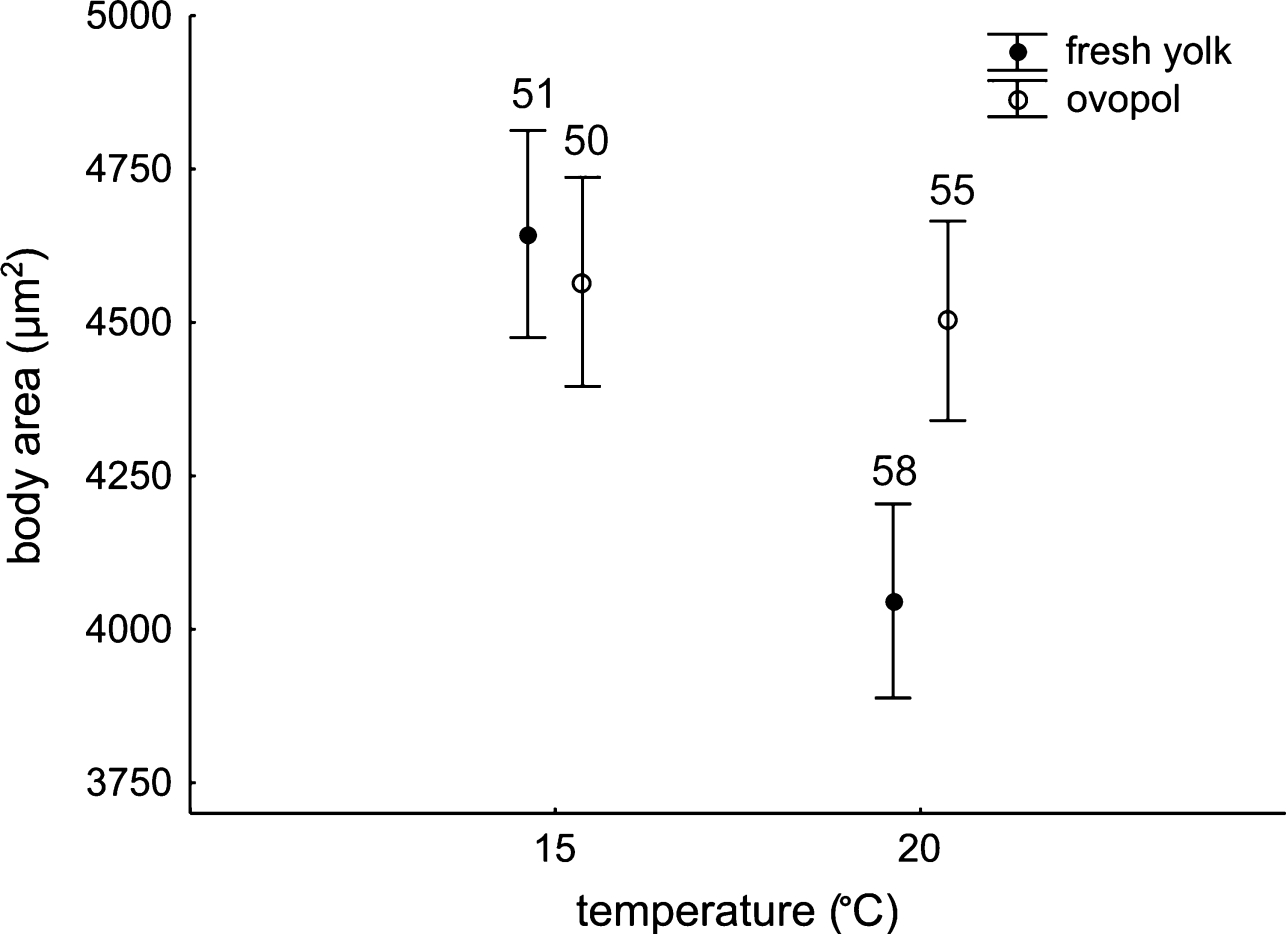
Body size of *Lecane inermis* at two temperatures in a laboratory experiment. GLM with temperature (fixed) and food type (random) as predictors (means ± 95% CI). Numbers given are the sample sizes.

We performed a GLM analysis on the entire dataset and for two factors, temperature (fixed) and food type (random). The main effects were not significant (temperature: F(1, 210) = 1.53, *P* = 0.432; food type: F(1, 210) = 0.50, *P* = 0.608), but there was a significant interaction effect (F(1, 210) = 10.19, *P* = 0.002). Rotifers decrease in size with increasing temperature when fed on fresh yolk only (post hoc Tukey test; Fig. 6). The length of an outstretched rotifer was more responsive to temperature (GLM model: *P* < 0.001 for temperature and *P* = 0.528 for food type), and the width was more responsive to food type (*P* = 0.044 for temperature and *P* < 0.001 for food type).

### Body size – sludge study

**Figure 7:**
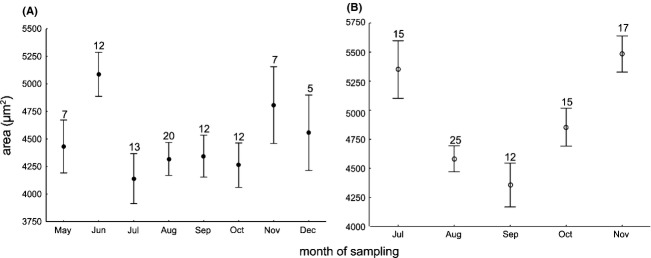
The seasonal variation of the body size of two rotifer species from activated sludge: *Lecane inermis* (A) and *Cephalodella gracilis* (B); means ± SE. Numbers given are the sample sizes analyzed per sampling.

During the year, *L. inermis* rotifers were observed only in samples taken from May to December. The remainder of the year likely had temperatures that were too low, resulting in populations below detection limit. The sizes of *L. inermis* differed significantly across months (F(7, 80) = 2.18; *P* = 0.045; Fig. 7a), as did those of *C. gracilis* (F(4, 79) = 7.89; *P* < 0.001; Fig. 7b).

**Figure 8:**
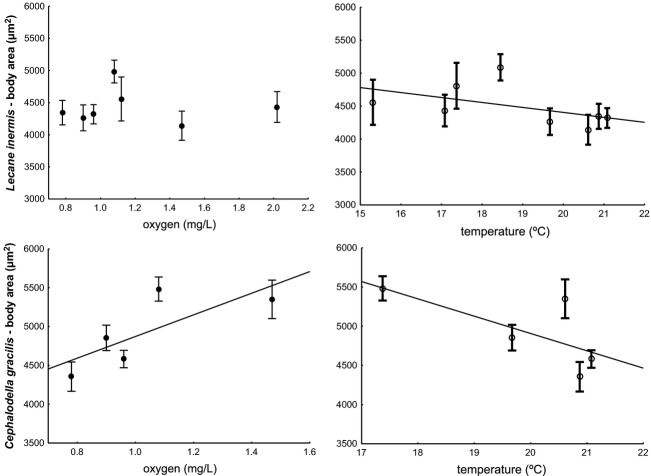
Relationship of the size of two rotifer species to temperature and oxygen (monthly means). The linear fit illustrates the relationships, which were found significant in multiple regression analyses for each species independently. Means ± SE.

A multiple regression on the body size response to temperature and oxygen content in activated sludge showed that for both measures of the parameters, mean and mid-mean values, the sizes of *L. inermis* depended significantly on temperature (*P* = 0.015 for monthly means and *P* = 0.011 for mid-monthly means), but not on oxygen (*P* = 0.224 for monthly means and *P* = 0.135 for mid monthly means). In the case of *C. gracilis*, the seasonal variability in body size was explained by both temperature and oxygen, regardless of the measure used (temperature: *P* < 0.001 for both mean measures; oxygen: *P* < 0.001 for monthly means and *P* = 0.007 for mid- monthly means). The significant relationships with temperature are in accordance with TSR predictions (Fig. 8). The significant relationship between *C. gracilis* body size and oxygen level supports the hypothesis that oxygen may be the ultimate driver of the TSR relationship.

## Discussion

We found that the seasonal variability in the body sizes (measured as the area of live organisms) of two monogonont rotifer species in activated sludge, *L. inermis* and *C. gracilis*, was significantly related to temperature. The direction of the changes (i.e., the decrease in size with increasing temperature) was consistent with Temperature-Size Rule predictions (Fig. 8). Moreover, in one of the species investigated, *C. gracilis*, this variability was also significantly related to oxygen content (Fig. 8). These two parameters, temperature and oxygen content, were also selected by PCA analysis as the most important parameters in shaping the properties of activated sludge, along with SVI and pH (Table 4, Fig. 5). However, SVI was significantly positively correlated with oxygen and pH was significantly positively correlated with temperature (Table 3). Therefore, their potential role in a pattern we tested is not distinguishable from the effects of oxygen and temperature.

So far, TSR has been demonstrated in several rotifer species: *Anuraeopsis fissa* and *Brachionus angularis* (Galindo et al. 1993), *Keratella cochlearis* (Bielańska-Grajner 1995; Green 1998), *Synchaeta pectinata* (Stelzer 2002), *Keratella tropica* (Gilbert 2011), *Brachionus calyciflorus* (Sun and Niu 2012), and *Brachionus plicatilis* (Walczyńska and Serra 2014). In the present study, we not only extend this list, but we also indicate some important factors that may affect the size–temperature relationship.

One of these factors was food quality. In our laboratory study, we observed a general decrease in size with increasing temperature in *L. inermis*, but the pattern was complicated by an interaction with food conditions. Rotifers decreased markedly in size with increasing temperature when fed on fresh yolk, while for those fed on ovopol, the difference in size with temperature was not statistically detectable (Fig. 6). This result is in accordance with another theory explaining TSR, the supply-demand theory, stating that temperature interacts with resources to set body size, because “an organism should continue to grow until it uses as much energy as it can reliably use on average, and no more” (DeLong 2012). Similarly, the poor food conditions could act as a limitation for following plastic growth pattern in accordance with TSR, because phenotypic plasticity is costly (Relyea 2002; DeWitt and Langerhans 2004). The latter would assume the restricting conditions in our ovopol treatment. From our experience, this type of food is good enough to keep rotifers in a good condition culture, yet, we did not control for its quality during the study.

The possibility that food conditions may alter the response of body size to temperature in rotifers had been previously raised by Galindo et al. (1993), and Wojewodzic et al. (2011). The authors of the latter even found that food limitation was more important than temperature in determining the body size of *Brachionus calyciflorus*. We found that the length of an outstretched rotifer was more responsive to temperature, while the width was more responsive to food type.

Another issue potentially affecting the TSR pattern is the distinction between its proximate and ultimate mechanisms. It was previously suggested that temperature was only a proximate factor, a simple response cue, while another covarying parameter might be the ultimate factor. One candidate for this ultimate factor is oxygen availability (dissolved oxygen concentration naturally decreases with increasing temperature while oxygen demand increases; Woods 1999; Atkinson et al. 2006; Harrison et al. 2013). In activated sludge, this relationship is externally controlled, which enabled us to disentangle it from the temperature effect. During sampling season, we found for one of the studied species, *C. gracilis*, a significant decrease in size with increasing temperature, but increase in size with increasing oxygen (Fig. 8, lower panels), which supports the prediction on adjustment to smaller size at lower oxygen concentration at higher temperature (Woods 1999).

*C. gracilis* showed a seasonal variation in size as a response to both temperature and oxygen, whether calculated as a monthly mean or as the mean of the mid-periods between samplings. We did not find a relationship between body size and oxygen content in *L. inermis*. However, such a relationship was previously reported in a laboratory study on two levels of temperature and oxygen (A. Walczyńska et al., submitted data). Our results do not indicate why we did not find this dependence in activated sludge. According to Figure 7, the size of *L. inermis* in June (the highest value for a whole dataset) did not fit the general pattern, meaning that another, undetected factor could play a role. One such factor could be sludge age (MCRT). A shorter MCRT means a higher fraction of biomass removed from the system every day, which could act like extrinsic mortality to its resident organisms at a population level.

**Figure 9:**
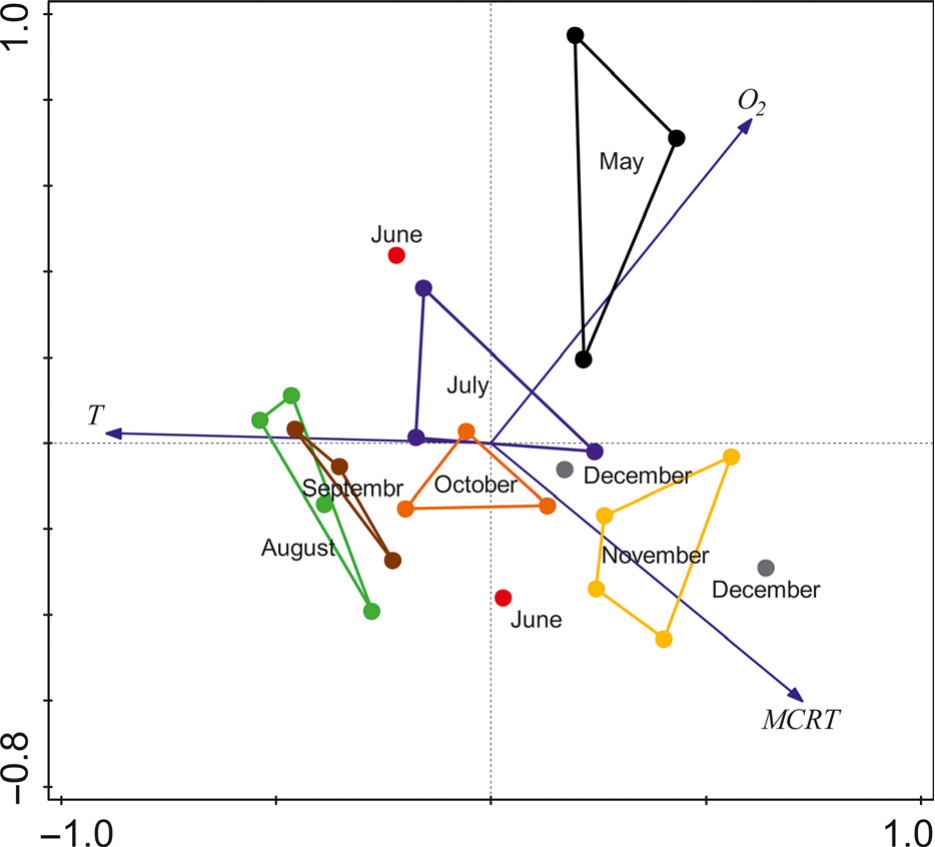
Biplot of the relationships among temperature, oxygen, and MCRT (PCA on *Z*-standardized values) merged with weekly means for each month from the period May–December 2012. Each point is one date of MCRT calculation within a season.

External mortality is a very important factor shaping an organism's life history (Kozłowski 1992; Roff 1992; Stearns 1992). The considerable effect of sludge age on the composition of the biological component of activated sludge has been previously shown by Pala-Ozkok et al. (2013). Unfortunately, in our study, this parameter was only estimated roughly, and at intervals of more than a week, so including it in a PCA analysis was not possible. However, for the sake of illustration, the PCA analysis for the common distribution of temperature, oxygen content, and MCRT was merged with weekly data of these parameters for the period May–December 2012 (Fig. 9). The figure shows that the grouping of data representing a particular month clustered with different parameters. For example, the data for May were most related to high oxygen values, which could explain the relatively large size of *L. inermis* for this month (Fig. 7) provided that size is oxygen-dependent. The data for November and December clustered with the higher values of MCRT, which acted as external mortality and explains the relatively large size of *L. inermis* for this period (Fig. 7). Interestingly, one data point for June was also associated with high values of MCRT, being consistent with our hypothesis that the extremely large *L. inermis* in June were the result of a higher sludge age (= low mortality). Finally, the summer months were most related to temperature, with comparable lengths of MCRT and relatively low oxygen. These factors were reflected in the relatively small size of *L. inermis* (Fig. 7). Overall, MCRT likely interacts with temperature to influence *L. inermis*' body size, and this relationship could be the reason why we did not detect a significant relationship between the body size and oxygen. The alternative scenario is the effect of resources, which appeared to play a role in TSR response in our laboratory study. Resources level was shown to drive temporal variation in body size in protists (DeLong et al. 2014) and vertebrates (Yom-Tov et al. 2007). Yet, we did not manipulate the food quality in the sludge during sampling period and we were unable to conclude on this issue.

Body size of *C. gracilis* almost perfectly tracked the values related to oxygen in Figure 9, with the largest animals appearing in July and November (highest oxygen values) and smallest appearing in August–September (lowest oxygen values; Figs. 9). This means that the two species we investigated differ in their sensitivity to sludge parameters: *C. gracilis* is much more sensitive to oxygen than *L. inermis*, and/or *C. gracilis* has shorter lifespan and is therefore less sensitive to sludge age than is *L. inermis*.

Another conclusion of our study relates to the best measure of the size of live rotifers. We found that body area predicts size differences better than lorica, although both measures were reliable. This might be especially important where food conditions differ, as highlighted above; measuring only the lorica may overemphasise differences associated with food conditions. On the other hand, we showed that toes are not a good measure of body size differences in *L. inermis* because their lengths did not change with the rotifer's size.

We assume that the age structure of rotifers was stable during sampling period, meaning that every time we sampled the individuals of similar age. This pattern can be disturbed by external mortality, causing the mean age and size in population to decrease. According to our results, it can affect *L. inermis* only, because *C. gracilis* appeared to be not sensitive to sludge age. On the other hand, the newly hatched *L. inermis* females are relatively large and do not differ very much in size in comparison with adults (egg size composes *c*. 45% of mother size; A. Walczyńska, personal data). We therefore argue that our analysis was not affected by the changes in the age structure.

Although we detected several local factors that underlie the dynamics of activated sludge, manipulative experiments are necessary to establish the causal relationships with the rotifer body size. Comparative studies in other WWTPs would help to find general patterns. To our knowledge, the only research where multivariate analyses were used to describe the processes in activated sludge is presented by Elissen et al. (2008). Their study took into account temporal and spatial patterns. Yet, their conclusions were rather general than circumstantial because of the high level of system complexity. Our study focuses on the local processes; thus, its more general applicability concerning internal properties of activated sludge would demand broader perspective. Nevertheless, we found strong support for ecological relationships we examined.

This study can potentially inform the control of the biological agent *Lecane inermis*. Because the use of *L. inermis* has been proposed as a tool for bulking control (Fiałkowska and Pajdak-Stós 2008), it is important to investigate the biology of this species in relation to temperature. The bulking and foaming phenomena are mainly caused by the filamentous bacteria *Microthrix parvicella,* which is common in WWTP with nutrient removal, especially when temperature in bioreactors drops below 15°C (Eikelboom 2000). Our results agree with earlier data presenting that life history of *L. inermis* is temperature-dependent (Fiałkowska et al. 2011), though previously the population parameters (population growth rate and population doubling time) were analyzed. We now specify this finding by showing that on the level of individuals, the temperature dependence of *L. inermis* is in accordance with TSR.

Pajdak-Stós and Fiałkowska (2012) showed that rotifers are effective in limiting how filamentous bacteria *M. parvicella* and *N. limicola* increase with temperature. Such an effect is most likely caused by their higher growth rates in higher temperatures and the rotifers' density-dependent influence on the abundance of filaments. However, taking into account the plasticity in the rotifers' size as a response to temperature change, one cannot exclude the possibility that rotifers are equally effective at ingesting filamentous bacteria per individual at lower temperatures. The relationship between *L. inermis* size and oxygen content in activated sludge requires further studies. We did not find the relationship, despite the support from a laboratory study (A. Walczyńska et al., submitted data) and despite the significant relationship found in another rotifer species. Providing that the existence of such a relationship is supported, the management implication for *L. inermis* as a bulking control agent would be that sludge should be aerated in winter because larger size at lower temperature (possible proximate factor) is an anticipation of high oxygen content (possible ultimate factor). Imitating the natural negative relationship between these two parameters may cause a proliferation of *L. inermis* population. Such a procedure would be relatively easy to apply and could maximize the effectiveness of certain WWTP.

## Conclusions

In our study on a seasonal variation of body size in two rotifer species living in a seminatural, dynamic environment (activated sludge), we showed that (1) both species follow the Temperature-Size Rule, (2) one of the species, *C. gracilis*, responds also to oxygen content, supporting the hypothesis on the ultimate role of oxygen for TSR, and (3) another species, *L. inermis*, does not significantly respond to oxygen, but we suggest that this effect is masked by the species sensitivity to sludge age, acting as external mortality factor. To our knowledge, this is the first study where the seasonal TSR pattern was demonstrated. Additionally, we found in a laboratory study that *L. inermis* performs TSR depending on food type, which raises the new questions on the role of resources in TSR.
